# A technical note on autologous iliac crest bone grafting for restoration of the distal tibial articular surface

**DOI:** 10.1186/s40634-023-00612-0

**Published:** 2023-04-30

**Authors:** Bahaa Zakarya Hasan, Mohamed Kamal Mesregah

**Affiliations:** Department of Orthopaedic Surgery, Menoufia University Faculty of Medicine, Shebin El-Kom, Menoufia Egypt

**Keywords:** Giant cell tumor, Distal tibia, Curettage, High-speed burr, Iliac crest grafting, Articular surface reconstruction

## Abstract

**Purpose:**

This technical note describes a reconstructive technique of the distal tibial articular surface using autologous iliac crest bone graft.

**Methods:**

Following curettage and high-speed burring of giant cell tumor of bone (GCTB) of the distal tibial articular surface, the resulting cavity was filled, and the articular surface was reconstructed using autologous tricortical iliac crest bone graft. The graft was fixed to the tibia with a plate.

**Results:**

The smooth congruent articulating surface of the distal tibia was restored. Full ankle range of motion was achieved. No recurrence was detected in the follow-up imaging.

**Conclusions:**

The currently reported technique using autologous tricortical iliac crest bone graft is a viable option for reconstructing the articular surface of the distal tibia.

## Introduction

Giant cell tumor of bone (GCTB) is a benign locally aggressive tumor representing 5% of all primary bone tumors [22]. This tumor rarely metastasizes, but it has a high tendency for local recurrence [10, 20].

GCTB usually occurs in patients aged 20–40 and commonly involves the epi-metaphysis of long bones and may compromise the articular surface integrity [7, 9].

The distal femur, proximal tibia, distal radius, and sacrum are the most common sites of GCTB [3]. Foot and ankle involvement with GCTB is uncommon, accounting for less than 4% of all GCTBs [15].

Treatment of GCTB should aim for local control without sacrificing joint function [9, 21]. The functional preserving surgery for GCTB is extended curettage with high-speed burring and chemical adjuvants such as liquid nitrogen, alcohol, or phenol and filling the resulting cavity with polymethylmethacrylate (PMMA) bone cement, bone substitutes, or bone graft [9, 18, 20].

In advanced cases where joint salvage is not feasible, en-bloc resection and endoprosthetic reconstruction may be an option but may result in increased morbidity and unfavorable functional outcomes in GCTB patients, who are frequently young and active [11, 19].

The incidence of GCTB in the distal tibia is rare [13, 15]. Management of tumors of distal tibia after curettage or resection varies depending on plenty of influencing factors, and the reported options include allograft reconstruction, ankle fusion or endoprosthetic reconstruction [1, 16, 17].

This article reports a reconstructive technique for the ankle joint after curettage and high-speed burring of GCTB using an autologous iliac crest bone graft to reconstruct the articulating part of the distal tibia.

### Operative technique

The technique presented in this article was approved by our Institutional Review Board (IRB) (IRB number: ORTH14-2) for reconstructing the ankle joint with an autologous tricortical iliac crest bone graft after curettage of GCTB of the distal tibia destructing the articular surface.

### 1-Preoperative evaluation

**Fig. 1:**
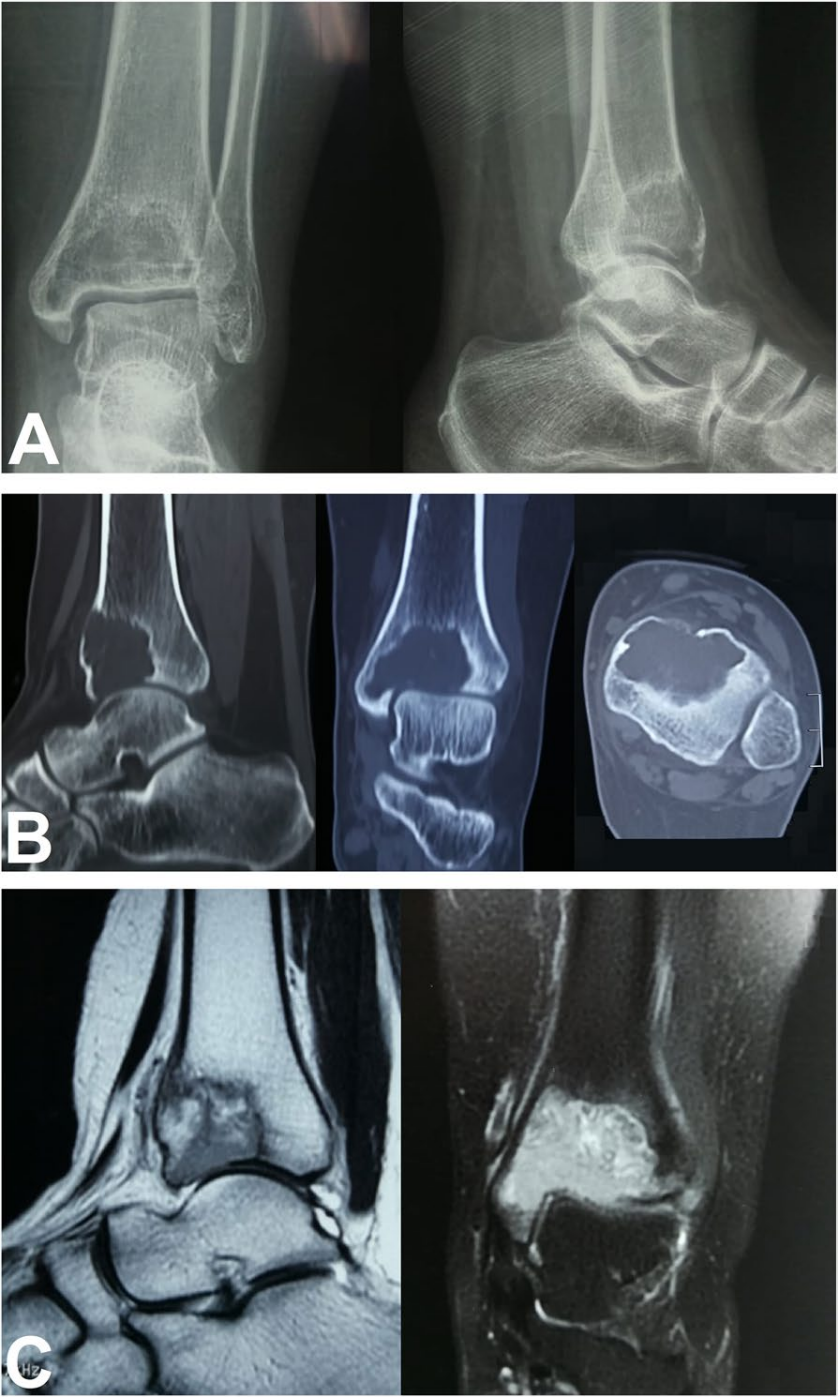
A 22-year-old female presented with limping, pain aggravated on walking, and restricted movement of the left ankle for 7 months. The patient had no history of trauma and no constitutional symptoms. Examination revealed tenderness over the anterior aspect of the distal tibia, with painful and restricted range of motion of the left ankle. **A** Preoperative anteroposterior and lateral X-rays showing a well-defined expansile osteolytic lesion in the epi-metaphyseal region of the left distal tibia suggestive of GCTB. The tumor was classified as Campanacci grade III. **B** Sagittal, coronal and axial CT scans showing an osteolytic lesion occupying the anterior two-thirds of the epi-metaphyseal region of the left distal tibia with erosion of the articular surface. **C** Sagittal and coronal MRI images showing an eccentric expansile osteolytic lesion measuring 4 (craniocaudal) × 3.5 (transverse) × 2.5 (anteroposterior) cm breaching the articular surface with only the posterior one-third of the tibial plafond preserved with no soft tissue or intra-articular extension

Proper history taking is crucial, including the duration of symptoms. General and local ankle examinations should be performed, including examination of the swelling if present, the site of tenderness and ankle range of motion. Imaging studies in the form of ankle X-rays, computed tomography (CT), and magnetic resonance imaging (MRI) are done for provisional diagnosis and proper assessment of tumor characteristics, Fig. 1. The size and extent of the osteolytic lesion should be precisely measured in the CT and MRI with the evaluation of cortical breach and extra-osseous tissue extension. Tumors should be classified using the Campanacci radiographic grading [5]. CT-guided biopsy should be obtained to confirm the diagnosis of GCTB.

### 2-Set‑up

The patient is placed supine under spinal anesthesia. Prophylactic 3^rd^ generation cephalosporin antibiotic should be administrated, and an above-knee tourniquet should be used.

### 3-Surgical approach

Sterile precautions and careful soft tissue handling are crucial. A standard anterior ankle approach is performed. A midline anterior longitudinal incision is made over the ankle. Subcutaneous tissue is dissected, and the extensor retinaculum is incised in line with the skin incision. The extensor hallucis longus tendon and the neurovascular bundle are retracted medially, and the extensor digitorum longus tendon is retracted laterally. The joint capsule is then opened. Bone is exposed, and a cortical window is made if the cortical bone is intact.

### 4-Curettage and high-speed burring

Curettage is done by different-sized bone curettes. A high-speed burr is then used to extend the curettage beyond the tumor margin. A thorough wash with H_2_O_2_ and saline solutions is done. After curettage, the resulting cavity should be measured in order to obtain a matched-sized tricortical iliac crest bone graft.

### 5-Tricortical iliac crest bone graft harvesting

Ipsilateral tricortical iliac crest bone graft is harvested in the usual manner. The graft size should be based on the size of the defect, and it must be large enough to fully reconstruct the bone defect. After appropriate size calculation, the bone graft is cut using an oscillating saw.

### 6‑Reconstruction of the distal tibial defect

**Fig. 2:**
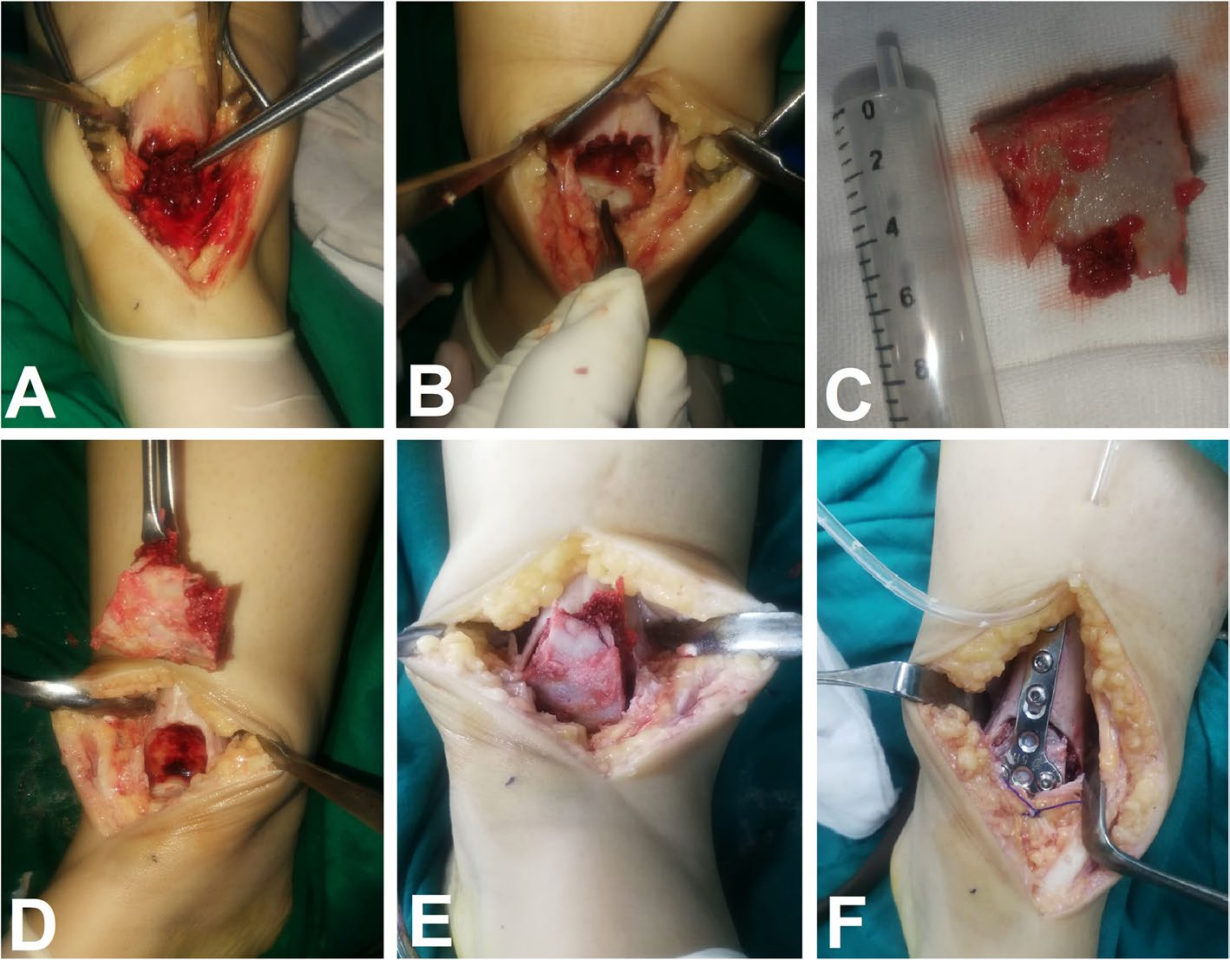
Intraoperative photographs **A**) Curettage of the lesion using different-sized curettes. **B** The cavity after curettage and high-speed burring with exposed articular cartilage of the talus. **C** The harvested tricortical iliac crest autograft. **D** Testing the shape and size of the graft in relation to the defect. **E** Placing the graft in the defect after trimming and fashioning. **F** Fixation of the graft to the tibia using a distal radius plate

The tricortical iliac crest bone graft is then prepared and fashioned using a bone nibbler to adapt to the cavity and the articular surface. The graft is placed and impacted in the cavity in a reversed manner so that the iliac crest cartilaginous cap reconstructs the articular surface. A space of 2 mm is left between the graft and the articular cartilage of the talus. A suitable-sized anterior plate, such as a distal radial plate, is then used to fix the graft to the tibia, Fig. 2. C-arm photos are obtained. A drain is placed, and the wound is closed. The specimen should be sent to histopathology for further examination.

### 7-Postoperative follow-up and rehabilitation

**Fig. 3:**
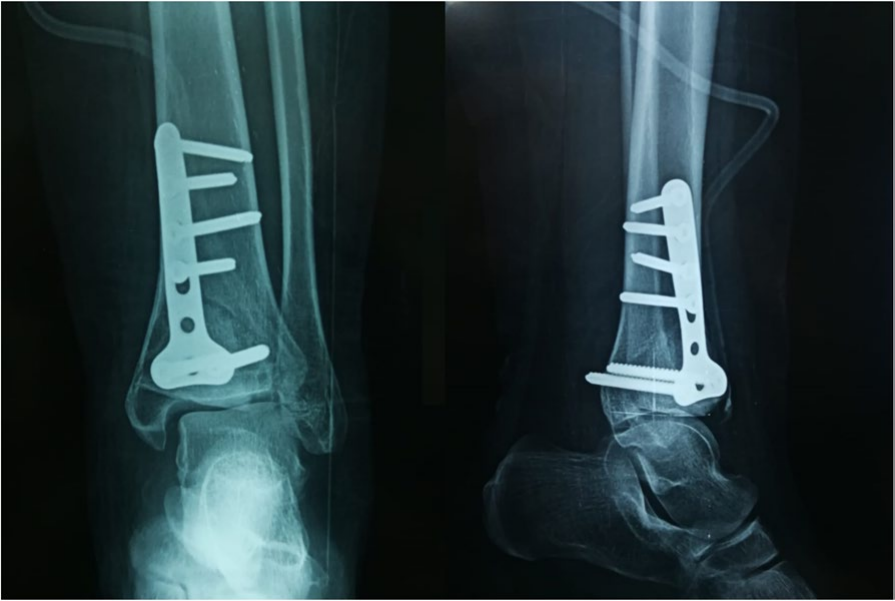
Immediate postoperative anteroposterior and lateral X-rays

The operated leg should be placed in a below-knee splint. Immediate postoperative ankle X-rays are obtained, Fig. 3. The drain is removed after 24 h. The splint and the stitches should be removed in 2–3 weeks. Thereafter, the patient is instructed to perform ankle flexion–extension range of motion exercises, with no weight bearing for the first 6 weeks postoperatively. Partial weight bearing as tolerated is allowed in the second 6 weeks postoperatively. Full weight bearing should commence 3 months postoperatively.

**Fig. 4:**
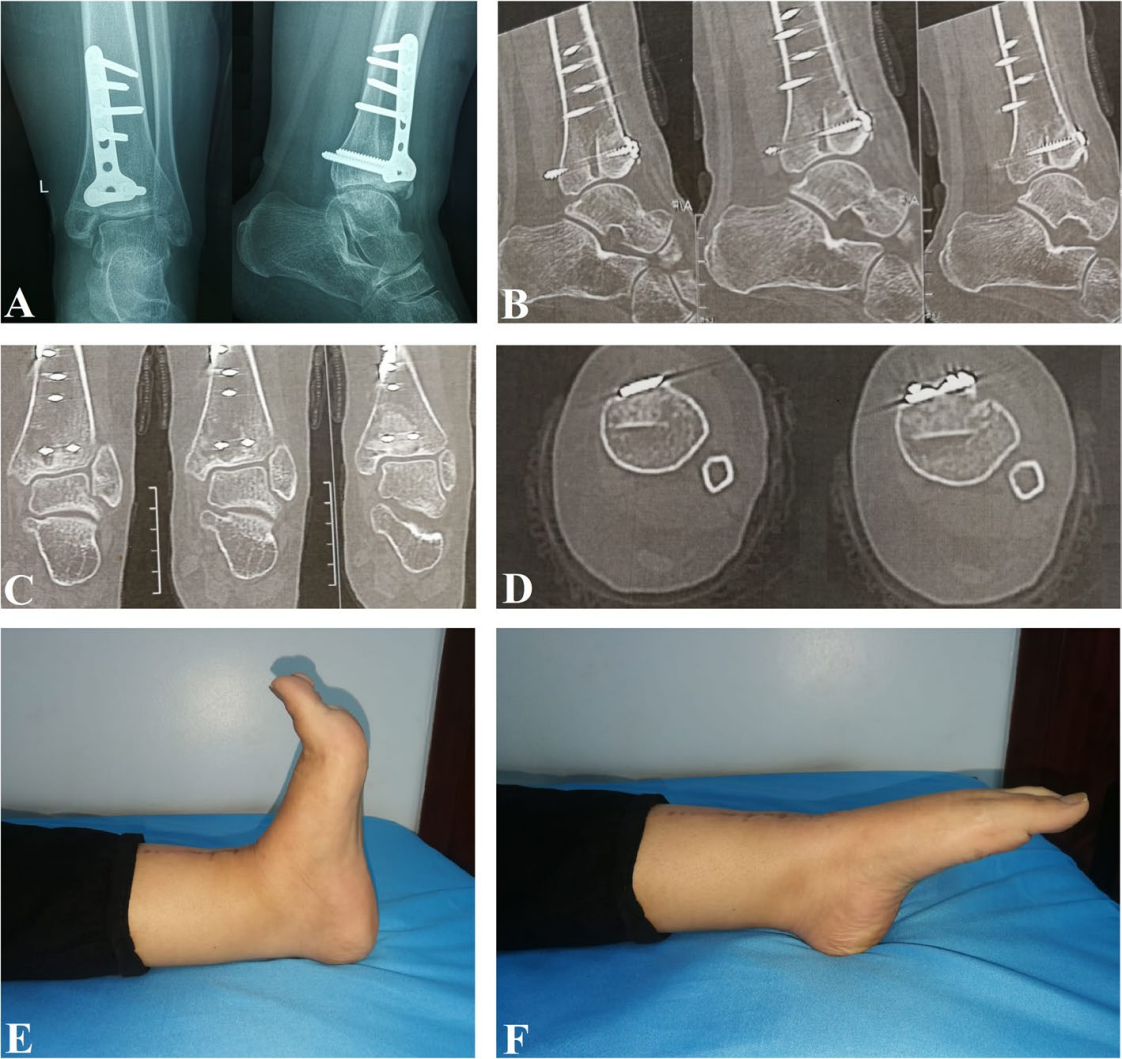
Six months follow-up radiological images showing complete union of the graft and reconstruction of the distal tibial articular surface. No donor site morbidity symptoms were reported. Clinical photos showing pain-free full range of motion of the ankle joint. **A** Anteroposterior and lateral plain X-rays. **B** Sagittal CT cuts. **C** Coronal CT cuts. **D** Axial CT cuts. **E** Ankle dorsiflexion. **F** Ankle plantar flexion

**Fig. 5:**
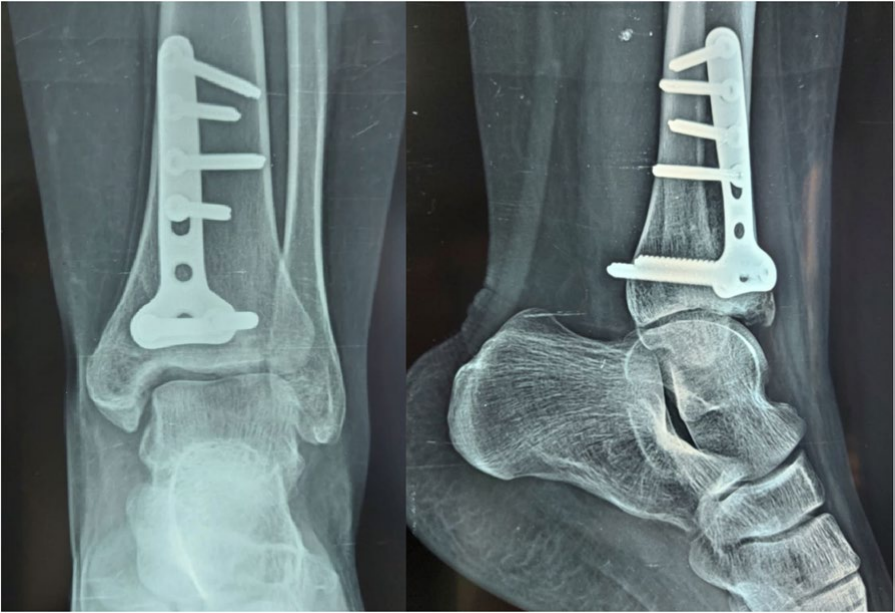
Fifteen months anteroposterior and lateral plain X-rays showing no signs of local recurrence

Regular follow-up visits should include clinical examination and ankle X-rays and CT scans to evaluate the union of the graft and the reconstruction of the articular surface and to detect any tumor recurrence, Figs. 4 and 5. It is recommended to have X-rays every 6 months for the first 2 years, and then annually till 5 years.

## Discussion

GCTB should be considered a differential diagnosis in all lytic bone lesions. While GCTBs usually occur in the distal femur, the proximal tibia, the distal radius, and the sacrum, they can also occur in rare locations, such as the distal tibia [3, 15].

Management of GCTB of the distal tibia is challenging, especially for tumors encroaching on the articular surface [15]. Moreover, having inadequate soft tissue coverage in the ankle may increase the risk of poor wound healing, especially with multiple surgeries; therefore, it is essential to avoid recurrence [15].

Several management options are reported in the literature. In their retrospective study of 31 patients with GCTB of the distal tibia, AlSulaimani et al. [2] recommended extended curettage for adequate control of the tumor. Despite having a 29% rate of local recurrence, recurrences were manageable with repeated curettage [2].

Paul et al. [15] reported 8 (42.1%) cases with GCTB of the distal tibia out of 19 patients with GCTBs of the foot and ankle over 9 years, treated with excision and bone graft (*n* = 3), extended curettage and bone graft (*n* = 2), and excision and mega prosthesis (*n* = 2), extended curettage and bone cement (*n* = 1). Wound infection (*n* = 2) and chronic osteomyelitis (*n* = 1) were reported as complications in patients who had previous surgeries. No local recurrence was detected after the index surgery in a mean follow-up of 36.2 months [15]. Previous studies reported no recurrence following intralesional curettage with filling the cavity with bone cement in patients with GCTB of the distal tibia [4, 13, 14].

Cribb et al. [6] reported a technique of curettage, high-speed burring, and ankle stabilization using Ilizarov fixator and reported excellent functional results with no tumor recurrence. Saglik et al. [16] described distal tibial resection and ankle arthrodesis using fibular autograft and reported pain-free daily activities. Economopoulos et al. [8] described ankle arthrodesis using a custom-made porous tantalum spacer and reported pain-free walking and no recurrence. Wiratnaya [23] reported a technique of wide-margin resection followed by tibialisation of fibula and ankle arthrodesis as an alternative option, with good functional outcomes and with no complications over 2 years of follow-up.

However, the drawback of ankle arthrodesis is the elimination of the ankle range of motion which is critical in those active young patients in their work years [17]. Moreover, the endoprosthetic replacement of the ankle joint has unpredictable long-term outcomes and implant survival [1].

Osteoarticular allograft reconstruction is another reported option, but it has drawbacks, including infection, graft lysis, and osteoarthritis [17, 24].

In the case of articular surface destruction, an autologous tricortical iliac crest bone graft can be used with the technical considerations reported in this technical note. This technique is an excellent option to fill the cavity and achieve anatomical restoration of the articular surface while preserving the ankle range of motion. Furthermore, curettage and high-speed burring and filling the resulting cavity with autografts give excellent results and reduce the chances of recurrence. The drawbacks of using autografts include donor site morbidity such as infection, residual scar, pain, or sensory changes [12]. Additionally, there is difficulty in detecting recurrence in X-rays with grafts in place. Further clinical studies are needed to validate the long-term efficacy and functional outcomes of the current technical note.

## Conclusion

In the setting of GCTB destroying the articular surface of the distal tibia, the current technical description of autologous tricortical iliac crest bone graft is sufficient to fill the resulting cavity after curettage and high-speed burring and could restore the smooth congruent articulating surface of the distal tibia, with preservation of the range of motion.

## Data Availability

The dataset used in this study is available from the corresponding author on request.
